# Human myiasis in Ecuador

**DOI:** 10.1371/journal.pntd.0007858

**Published:** 2020-02-21

**Authors:** Manuel Calvopina, Esteban Ortiz-Prado, Byron Castañeda, Isabel Cueva, Richar Rodriguez-Hidalgo, Philip J. Cooper

**Affiliations:** 1 OneHealth Research Group, Facultad de Ciencias de la Salud, Universidad De Las Américas (UDLA), Quito, Ecuador; 2 Department of Cell Biology, Physiology and Immunology, Universitat de Barcelona, Barcelona, Spain; 3 Institute of Tropical Medicine and International Health, Charité – Universitätsmedizin, Berlin, Germany; 4 Instituto Superior Tecnológico, Cruz Roja Ecuatoriana, Quito, Ecuador; 5 Instituto de Investigación en Salud Pública y Zoonosis - CIZ, Universidad Central del Ecuador, Quito, Ecuador; 6 Facultad de Medicina Veterinaria y Zootecnia, Universidad Central del Ecuador, Quito, Ecuador; 7 Facultad de Ciencias Medicas, de la Salud y la Vida, Universidad Internacional del Ecuador, Quito, Ecuador; 8 Institute of Infection and Immunity, St. George’s University of London, London, England; Faculty of Medicine of University of Colombo, SRI LANKA

## Abstract

We review epidemiological and clinical data on human myiasis from Ecuador, based on data from the Ministry of Public Health (MPH) and a review of the available literature for clinical cases. The larvae of four flies, *Dermatobia hominis*, *Cochliomyia hominivorax*, *Sarcophaga haemorrhoidalis*, and *Lucilia eximia*, were identified as the causative agents in 39 reported clinical cases. The obligate *D*. *hominis*, causing furuncular lesions, caused 17 (43.5%) cases distributed along the tropical Pacific coast and the Amazon regions. The facultative *C*. *hominivorax* was identified in 15 (38%) clinical cases, infesting wound and cavitary lesions including orbital, nasal, aural and vaginal, and occurred in both subtropical and Andean regions. *C*. *hominivorax* was also identified in a nosocomial hospital-acquired wound. Single infestations were reported for *S*. *haemorrhoidalis* and *L*. *eximia*. Of the 39 clinical cases, 8 (21%) occurred in tourists. Ivermectin, when it became available, was used to treat furuncular, wound, and cavitary lesions successfully. MPH data for 2013–2015 registered 2,187 cases of which 54% were reported in men; 46% occurred in the tropical Pacific coast, 30% in the temperate Andes, 24% in the tropical Amazon, and 0.2% in the Galapagos Islands. The highest annual incidence was reported in the Amazon (23 cases/100,000 population), followed by Coast (5.1/100,000) and Andes (4.7/100,000). Human myiasis is a neglected and understudied ectoparasitic infestation, being endemic in both temperate and tropical regions of Ecuador. Improved education and awareness among populations living in, visitors to, and health personnel working in high-risk regions, is required for improved epidemiological surveillance, prevention, and correct diagnosis and treatment.

## Author summary

Human myiasis is a neglected disease caused by ectoparasitic infestations of the skin, cavities and wounds by larvae of a wide variety of dipteran flies. Here, we review available data on human myiasis in Ecuador using a national registry of 2,187 cases and other information sources (39 clinical cases). We conclude that myiasis is endemic in both temperate and tropical regions of the country with greatest endemicity in the tropical lowlands of the Amazon (estimated annual incidence of 23 cases/100,000 population). Almost half clinical cases were cutaneous furuncular myiasis caused by *Dermatobia hominis*, which produced single lesions that were treated successfully with oral ivermectin followed by manual extraction. Also relatively common were infestations by *Cochliomyia hominivorax*, found in tropical and temperate regions in the Andean highlands, which infested wounds and cavities, occasionally involving hundreds of larvae and causing severe tissue damage when unrecognized. Our data shows that myiasis is a significant and unrecognized health problem in Ecuador, is likely to be significantly under-reported, and requires improved awareness among affected populations and improved education among health professionals for its diagnosis, treatment, and recognition of fly species.

## Introduction

Human myiasis is an ectoparasitic infestation of living or dead tissues by larvae or maggots of several species of flies of the order Diptera, and together with the ectoparasitic scabies and tungiasis, is classed as a neglected tropical disease [1]. Human myiasis has a worldwide distribution but is more frequently reported from tropical regions, determined by the presence and geographic distribution of different fly species [2]. Ecuador is considered to be endemic for myiasis, particularly that caused by larvae of *Dermatobia hominis* that produce the furuncular clinical form [3].

**Fig 1:**
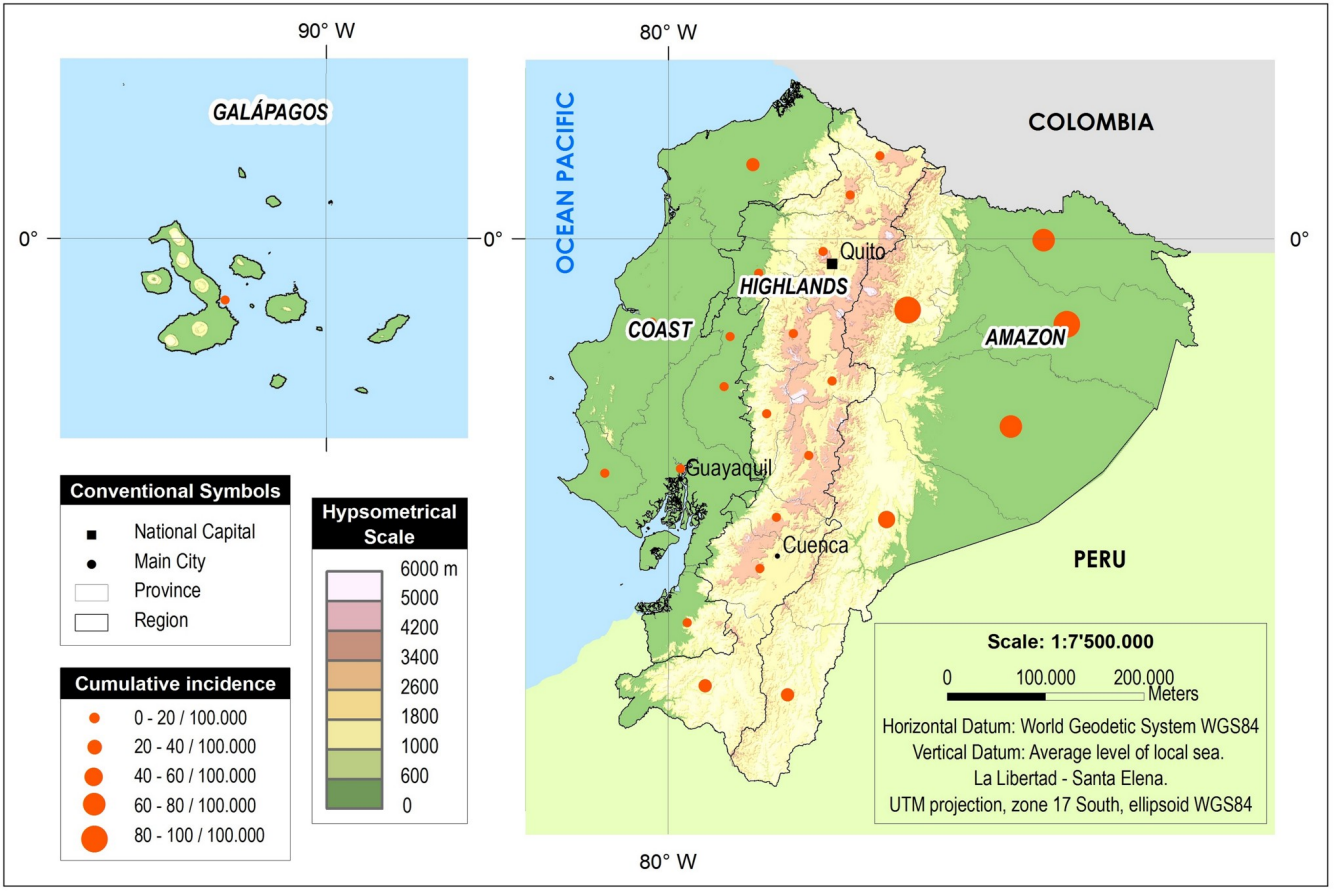
Map of Ecuador. Geographical distribution of the Ecuadorian Ministry of Public Health (MPH) registered cases per 100.000 inhabitants between 2013 and 2015. Data source RDACCA-Ecuadorian MPH. Figure made using ArcGIS software.

Ecuador, located in the Western Pacific region of South America, lies on the Equator and is crossed by the Andes mountain range, thus dividing the country into distinct geoclimatic regions: western Pacific coastal with subtropical and tropical lowlands, central Andean with high mountains and deep valleys where climates may be temperate to subtropical, and eastern Amazon lowlands of humid tropical rain forest (Fig 1). In addition, the Galapagos Islands, 1,000 km off the mainland in the Pacific, are part of Ecuador [4]. Tropical and subtropical regions cover approximately 64% of Ecuador’s landmass of 276,841 km^2^. The total population of Ecuador in the 2010 census was 14,483,499 of which 53% live in the Pacific coastal, 42% in Andean, 5% in the Amazon region, and 0.2% in the Galapagos Islands [5].

The dipterous larvae that cause myiasis include obligate (those feeding on live host tissue), facultative (decomposing tissues), and accidental (or pseudomyiasis) through ingestion of eggs or larvae from contaminated food or water. Myiasis may also be differentiated by the anatomical site of infestation: cutaneous (furuncular lesions and migratory or creeping dermal eruption); wound or traumatic causing cavitary lesions affecting the mouth (palate and periodontal), nose, nasopharynx, ear, eye (external and internal), anus and vagina; and enteric myiasis. Obligate myiasis can be serious or even fatal arising from complications relating to feeding of larvae on healthy tissues. Facultative myiasis may result in considerable pain and tissue damage while accidental myiasis is considered benign. Nosocomial myiasis refers to infestations that occur in a hospital setting [2,6].

Numerous fly species can cause human myiasis. The main species involved in obligate myiasis is *D*. *hominis*, which can be found from Mexico through Central and South America. Larvae feed under the skin, causing furuncular lesions with a typical air-pore. The main species causing facultative myiasis in the New World is *Cochliomyia hominivorax* (the New World screwworm). The *Oestridae* family of flies, a common cause of myiasis in sheep in Ecuador [7] have been associated with human infestations affecting eyes and nasopharyngeal cavities while accidental or pseudomyiasis has been associated with *Syrphidae*, *Stratiomyidae*, and *Tephritidae* families [8]. In Ecuador, four species of flies had been described to cause human myiasis: *D*. *hominis*, *Oestrus ovis*, *C*. *hominivorax* and *Sarcophaga haemorrhoidalis* [9].

Risk of myiasis is correlated with densities of fly populations and exposures through poor sanitation and personal hygiene, low socioeconomic status, and proximity to domestic animals. Elderly and seriously ill people, including those with severe intellectual disability and drug addiction are at higher risk. Neglected open wounds are also an important risk factor [10, 11]. Cutaneous myiasis is increasingly recognized among both tourist and business travelers to tropical regions of Central and South America [3].

There are limited data from Ecuador on the prevalence of human myiasis, geographic distribution, fly species involved, presence of different clinical forms, and management.

## Methods

### Ethics statement

The article is a review of published and publicly accessible anonymized material and required no human subjects ethics review.

We reviewed the published literature from local and international journals relating to human myiasis in Ecuador, as well as the official data from the National Automated Registry of Out-Patient Medical Consultations (RDACCA) database retrieved from the Ecuadorian Ministry of Public Health (MPH) repository. The main electronic libraries of PubMed, Scientific Electronic Library (Scielo), Latin American and Caribbean Health Sciences (LILACS), and Google Scholar were searched, using the following combination of terms in any given order,”myiasis and miasis”, “*Dermatobia hominis*”, “*Oestrus ovis*”, “*Cochlyomyia hominivorax*”, “*Sarcophaga haemorrhoidalis”*, “Ecuador”, with no restriction on language or publication date. Non-indexed local journals, bulletins, local meetings, abstracts, books, and clinical cases from personal collections and archives, were included in the review. RDACCA system uses International Classification of Diseases (ICD-10) to register cases. The information was retrieved searching for: B87 (Myiasis), B87.0 (Cutaneous myiasis), B87.1 (Wound myiasis), B87.2 (Ocular myiasis), B87.3 (Nasopharyngeal myiasis), B87.4 (Aural myiasis), B87.8 (Myiasis of other sites) and B87.9 (Myiasis, unspecified). All patients seen by the authors signed a consent form for publishing his/her case.

## Results

### Prevalence of infestation

National data were available for the three-year period, 2013 to 2015, during which 2,187 cases were registered by the MPH. We identified 39 clinical cases of human myiasis, 29 from the literature review [13–36] and 10 (31%) from our own clinical experience. Eight (21%) cases were reported among travelers from USA, Spain, Japan, Israel, Argentina and Germany. Thirteen of the 39 (41%) clinical cases were reported in international journals, 7 (22%) in Ecuadorian journals, 2 (6%) in non-indexed local publications. (Table 1).

**Table 1:** Summary of clinical case reports of human myiasis originating in Ecuador.

Reference Author/year (no. patients)	Age (years)	Gender	Ethnicity / Country of Origen	Classification: Biological / Clinical form	N° lesions (# larvae)	Anatomical localization	Fly spp. identification	Region	Treatment	Predisposing factors
Cooper Philip. 1991	23	M	White/ North American	Obligatory Furuncular	13 (13)	Back	*D*. *hominis*	Amazon	Occlusive dressings; surgical incision; antibiotic (cloxacillin)	Travelled to endemic area
Latorre M. et al., 1993 [13]	32	F	White/Spanish	Obligatory Furuncular	1 (1)	Limbs (shoulder)	*D*. *hominis*	NI	Surgical incision; antibiotic (cloxacillin)	Travelled to endemic area
Cooper Philip 1993	9	M	Afro-Ecuadorian/EC	Obligatory Furuncular	1 (1)	Face	*D*. *hominis*	Coast	Occlusive dressings; surgical incision	Lived in rural area
Westenfeld F, 1993 [14]	62	M	White/North American	Obligatory Furuncular	1 (1)	Limbs (elbow)	*D*. *hominis*	NI	Surgical incision	Travelled to endemic area
Cooper Philip 1993	6	M	Afro-Ecuadorian/EC	Obligatory Furuncular	1 (1)	Face/orbit	*D*. *hominis*	Coast	Surgical incision; antibiotic (cloxacillin)	Lived in rural area
Chico M. et al., 1994 [15]	30	M	Mestizo/EC	Obligatory Furuncular	3 (3)	Thorax (back)	*D*. *hominis*	Amazon	Surgical incision	Lived in rural area
	7	F	Indigenous Chachi/EC	Facultative Cavitary	1 (41)	Aural	*C*. *hominivorax*	Coast	Manual extraction	Lived in rural area
	0.9	M	Mestizo/EC	Facultative Cavitary	2 (3)	Aural orbital external	*S*. *haemorrhoidalis*	Coast	Manual extraction	Lived in rural area
Hosokawa A. et al., 2000 [16]	70	M	Mestizo/EC	Obligatory Furuncular	1 (1)	Limbs (elbow)	*D*. *hominis*	Andean	Surgical incision; antibiotics	Lived in rural area (farmer)
	7	M	Mestizo/EC	Obligatory Furuncular	1 (1)	Scalp	*D*. *hominis*	Andean	Manual extraction; topical antibiotics	Lived in rural area (farmer)
Ortega-Rosero J. et al., 2000 [17]	1	F	Mestizo/EC	Accidental	(1)	intestinal	*Callitroga americana* (Syn. *C*. *hominivorax*)	Coast	Self-elimination	Lived in rural area
Schwartz E. & Gur H. 2002 [18]	22	M	White/Israeli	Obligatory Furuncular	1 (1)	Limbs (leg)	*D*. *hominis*	NI	Surgical incision	Travelled to endemic area
Cabrera F. et al., 2002 [19]	21	M	Mestizo/EC	Facultative wound	1 (200)	Face	*C*. *hominivorax* *(D*. *hominis* by authors)	Coast	Intramuscular ivermectin, antibiotics, cryotherapy spray	Alcoholism, Lived in rural area
	63	M	Mestizo/EC	Facultative wound	1 (400)	Limbs (left leg)	*C*. *hominivorax* *(D*. *hominis* by authors)	Andean	Intramuscular ivermectin, antibiotics, manual extraction	Gangrenous pyoderma, Lived in rural area
Vaca-Aguirre P. 2003 [20]	73	M	Mestizo/EC	Facultative Cavitary	1 (multiple larvae)	Aural	NI	Coast	Antibiotics, surgical extraction	Myringoplasty, Lived in rural area
	25	M	Mestizo/EC	Facultative, Cavitary	1 (multiple larvae)	Aural	NI	Coast	Chloroform, antibiotics	Chronic otitis media, Lived in rural area
Guanga I. & Cruz C., 2006 [21]	59	F	Indigenous/Kichwa/EC	Facultative Cavitary	1 (40)	Nasal bilateral	*C*. *hominivorax*	Andean	Oral ivermectin, antibiotics, surgical extraction.	Alcoholism (died of CNS infection)
Andrade M. et al., 2007 [22]	2	F	Indigenous Kichwa/EC	Obligatory Furuncular	1 (1)	Limbs (Left thigh)	*D*. *hominis*	Amazon	Ceftriaxone, manual extraction	Contact with domestic animals
Nagamori K. et al., 2007 [23]	41	M	Asian/Japanese	Obligatory Furuncular	1 (1)	Thorax (left upper back)	*D*. *hominis*	NI	Surgical incision; antibiotics (levofloxacin, gentamycin)	Travelled to endemic area
Yerovi C. et al., 2008 [24]	82	F	NI	Facultative Cavitary	1 (multiple larvae)	Vagina	NI	Coast	Manual extraction	Vaginal verrucous carcinoma
Muller I. & Vitagliano G. 2011 [25]	30	M	White/Argentine	Obligatory Furuncular	1 (1)	Scrotum	*D*. *hominis*	NI	Surgical incision; antibiotic (cephalexin)	Travelled to endemic area
Avendaño A. et al., 2013 [26]	29	M	NI	Obligatory Furuncular	1 (NS)	Limbs (toe)	*D*. *hominis*	Coast	Manual extraction; antibiotics (amoxicilin/clavulanic acid, gentamycin)	Lived in rural area (farmer)
Angulo L. et al., 2014 [27]	22	F	White/Spanish	Obligatory Furuncular	11 (11)	Limbs (leg)	*D*. *hominis*	NI	Surgical incision	Travelled to endemic area
Cedeño J. et al., 2014 [28]	33	M	NI	Facultative Cavitary	1 (22)	Aural	*C*. *hominivorax*	Andean	Manual extraction; cigarette smoke; topical ivermectin; antibiotic (ciprofloxacin)	Alcoholism, farmer, living in rural area
Pinos VH. et al., 2014 [29]	75	F	Indigenous (Kichwa)/EC	Facultative Cavitary Wound	4 (> 300 larvae)	Nasal, Oral cavity, Aural, Orbital, Face	*C*. *hominivorax* *(D*. *hominis* by authors)	Andean	Surgical (enucleation both eyes); manual extraction; ivermectin; antibiotics (vancomycin, imipenem)	Burns, alcoholism, undernutrition
Alemán JM & Reinoso S. 2014 [30]	24	F	Mestizo/EC	Facultative Cavitary	3 (multiple larvae)	Oral Cavity	*C*. *hominivorax*	Andean	Surgical Incision; ivermectin	Chronic neurological deficit
Dominguez JP. et al., 2015 [31]	91	F	Indigenous (Kichwa)/EC	Facultative Cavitary	1 (>100 larvae)	Orbital	*C*. *hominivorax*	Andean	Manual extraction; antibiotics (ceftriaxone, metronidazole, tobramycin)	Elderly, mentally retarded, rural, poor hygiene & low socioeconomic status
Coronel AP. et al., 2016 [32]	90	F	NI	Facultative Wound	3 (multiple larvae)	Thorax (axillar), Vaginal and Anal	NI	Andean	Manual extraction, antibiotics (amikacin, clindamycin, vancomycin); ivermectin	Senile dementia
Piña-Tornés A. et al., 2016 [33]	30	M	NI	Obligatory Wound	1 (multiple larvae)	Scalp	*C*. *hominivorax* (*D*. *hominis* by authors)	Coast	Surgical incision; creolin; antibiotics (ciprofloxacin, metronidazole, ampicillin/sulbactam)	Indigent, schizophrenia
Dueñas O. et al., 2017 [34]	70	M	Mestizo/EC	Obligatory Wound	1 (1)	Thorax (left scapula)	NI	Andean	Manual extraction; antibiotic (cephalexin)	Elderly, lived in rural area
Ramirez JI. et al., 2017 [35]	0.4	M	NI	Facultative Wound (nosocomial)	1 (45)	Colostomy	*C*. *hominivorax*	Coast	Surgical incision; antibiotic (oxacillin)	Colostomy, anorectal malformation
Uslu U. et al., 2018 [36]	64	M	White/German	Obligatory Furuncular	2 (2)	Limbs (Forearm)	*D*. *hominis*	NI	Manual extraction; antibiotic (levofloxacin)	Travelled to endemic area Non-Hodgkin`s lymphoma
Cueva Isabel 2016	49	M	Mestizo/EC	Facultative Wound	1 (multiple larvae)	Limbs (leg)	*C*. *hominivorax*	Coast	Manual extraction; ivermectin; antibiotic (metronidazole)	Elephantiasis, low socio-economic status, lived in rural area
Cueva Isabel 2017	73	M	Mestizo/EC	Facultative Wound	1 (30)	Nasal, orbita, Face	*C*. *hominivorax*	Andean	Manual extraction; **i**vermectin	Basal cell carcinoma
Castañeda Byron 2015	73	M	Indigenous (Kichwa)/EC	Facultative Cavitary	1 (40)	Nasal	*C*. *hominivorax*	Andean	Mechanical aspiration; ivermectin	None
Castañeda Byron 2017	75	F	Indigenous (Kichwa)/EC	Facultative Wound	1 (15)	Limbs (Left leg)	*Lucilia eximia*	Andean	Manual extraction	Chronic renal failure
Calvopiña Manuel 2016	6	M	Mestizo/EC	Obligatory Furuncular	1 (1)	Face	*D*. *hominis*	Amazon	Ivermectin	none
Calvopiña Manuel 2018	21	F	Mestizo/EC	Obligatory Furuncular	1 (1)	Neck	*D*. *hominis*	Coast	Ivermectin	none
Calvopiña Manuel 2018	35	F	Mestizo/EC	Facultative Wound	1(41)	Nasal	*C*. *hominivorax*	Amazon	Manual extraction; ivermectin	Pituitary adenoma

NI, not identified. EC, Ecuador. [], reference numbers

### Geographical distribution

Of the 2,187 cases registered by the Ecuadorian MPH between 2013 and 2015, 1,006 (46%) were from the Pacific coastal region, 651 (30%) from temperate regions of the Andes, 525 (24%) from the Amazon region, and 5 (0.2%) from the Galapagos Islands. Using population data from the 2010 census, these data allow us to estimate the annual incidence as being highest in the Amazon (23 cases/100,000 population), followed by Coastal (5.1/100,000) and Andean (4.7/100.000) regions (Fig 1). Of the 39 clinical cases (from non-MPH registry sources), 13 (33%) were infested in the mountainous temperate zone of the Andes, 14 (36%) in the Pacific coastal region, and 5 (13%) from the Amazon region. Of the 8 (21%) cases among travelers to Ecuador, the geographic origin of infestation was only specified in 1 case (Amazon region).

### Age and sex

Among the MPH national official records (2013–2015) and the 39 clinical cases, myiasis was more frequent in men (54% and 64%, respectively). Among 2,187 registered cases by MPH, the proportion of cases was greatest among those of working age (i.e. 21–65 years) (Table 2). Among the 39 clinical cases, age ranged 4 months to 91 years (median, 30 years) with 51% occurring among those of working age (21–65 years), 26% in the elderly (i.e. >65 years), and 23% below 21 years of which 2 were infants (Table 1).

**Table 2:** Age group distribution for cases of myiasis reported in the Ecuadorian Ministry of Public Health registry (2013–2015) and for clinical cases identified from the literature search.

	Ministry of Public Health Registry Data		Literature Search Clinical Case Data	
Age group	(n)	%	(n)	%
**Pre-school (0–5 yrs)**	328	15	2	10.3
**School (6–12 yrs)**	247	11	3	12.8
**Adolescence (13–20 yrs)**	201	9	0	0.0
**Young adult (21–39 yrs)**	377	17	11	35.9
**Adult (40–65 yrs)**	383	18	4	15.4
**Elderly (>65 yrs)**	244	11	9	25.6
**Unknown**	406	19	0	0.0
**Total**	2186	100	39	100

### Biological classification

Almost half (49%) of clinical cases were obligatory and the other half (49%) were facultative. One case of accidental myiasis (or pseudomyiasis) was reported [35]. No biological status was identified among 2,187 registered cases from MPH. Data on biological classification (or fly species) were not available for the 2,187 MPH cases for which data were available only for clinical type (i.e. cutaneous, wound, etc.).

### Clinical lesions and fly species involved

**Fig 2:**
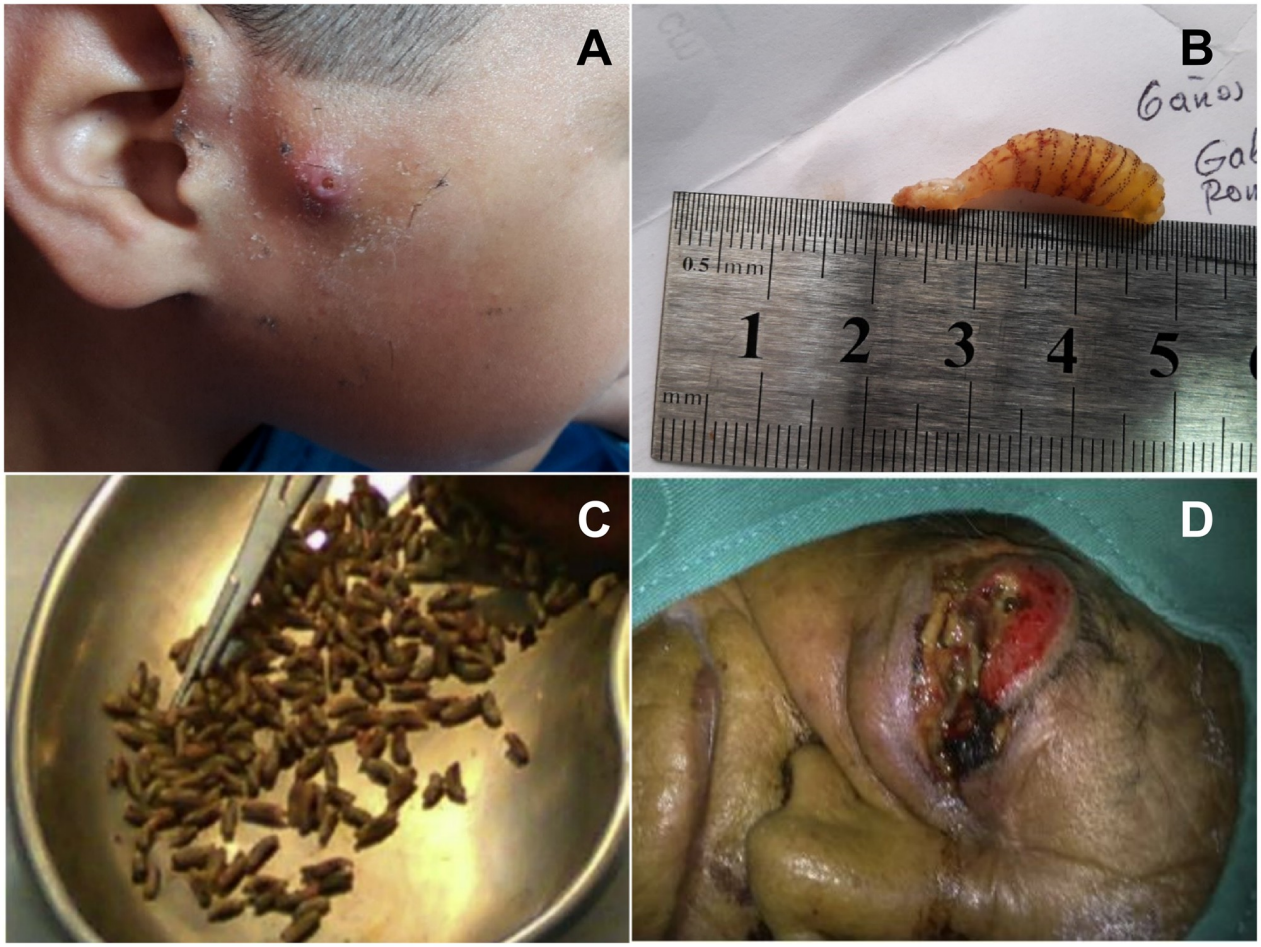
Furuncular and cavitary myiasis. Furuncular myiasis in a 6-years-old boy from the Amazon region showing a clinical lesion with central air pore (A)—a single larva was identified as *D*. *hominis* 3^rd^ instar (B). The child was treated with ivermectin (400 μg/kg once). After 48 h, a dead larva was extracted manually. More than 100 *C*. *hominivorax* 3^rd^ instar larvae (C) manually extracted from the right eyeball (D) of a 91 year-old indigent indigenous Kichwa woman. Infestation occurred in the Andes region at 2,418 m, 80 km from Quito, the capital.

Of a total of 70 lesions observed in the 39 clinical cases, 29% of lesions were localized to the head region, and 30% affected limbs, 3% genital area, 29% thorax, and 9% abdomen (Table 1). Furuncular lesions were found in 17 (42.5%) of reported cases, all caused by *D*. *hominis* (Fig 2A and 2B). In 7 wound and 7 cavitary clinical lesions, *C*. *hominivorax* was identified as the fly involved (Fig 2C and 2D). In single individuals with wound or cavitary lesions, the causative agents were identified as *Lucilia eximia* and *S*. *haemorrhoidalis*, respectively. Larvae were not formally identified in two patients with wound lesions and in three with cavitary lesions [20, 21, 24, 32, 34]. Larvae in 4 patients with wound lesions were mistakenly identified as *D*. *hominis* but in fact were *C*. *hominivora*x [19, 29, 33]. One wound case after colostomy was considered nosocomial being acquired during hospitalization and the larvae identified as *C*. *hominivorax* [35]. Of six cases in which larval instar was described, the second larval instar occurred in four cases. No fly species was identified among 2,187 registered cases from MPH.

Greater than a thousand larvae were extracted from the 70 lesions observed in the 39 clinical cases, varying from single larvae of *D*. *hominis* in each cutaneous furuncular lesion to 15 larvae of *L*. *eximia* in a case of wound myiasis to >100 larvae of *C*. *hominivorax* in wound and cavitary myiasis. Some cases had multiple furuncular lesions of *D*. *hominis*: for example, a tourist from USA had 13 lesions (Table 1). Clinical type and site of myiasis of the 2187 registered cases from MPH were stratified by age group are shown in Table 3. The proportion of clinical forms of myiasis by age did change across age groups. The relative frequency of cutaneous myiasis was greater at the extremes of age while wound myiasis was less frequent compared to other forms in young children.

**Table 3:** Clinical type by age group reported between 2013 and 2015 in the Ecuadorian Ministry of Public Health registry by International Classification of Diseases codings (ICD-10).

*Age group (years)*	*0 to 5*	*6 to 12*	*13 to 20*	*21 to 39*	*40 to 65*	*> 65*
*B87 Cutaneous myiasis*	45	32	19	36	54	33
*B87.1 Wound myiasis*	44	49	75	124	105	78
*B87.2 Ocular myiasis*	2	2	0	8	4	0
*B87.3 Nasopharyngeal myiasis*	3	0	0	2	0	1
*B87.4 Aural myiasis*	0	0	0	0	0	0
*B87.8 Myiasis of other sites*	13	6	1	7	8	5
*B87.9 Myiasis, unspecified*	289	226	174	269	280	193
*Total*	396	315	269	446	451	310

### Associated health conditions

Health conditions associated with clinical cases are detailed in Table 1. Myiasis occurred in 4 /39 (10%) of clinical cases diagnosed with cancer (basal cell carcinoma [wound myiasis/*C*. *hominivorax*, pituitary adenoma [wound myiasis/*C*. *hominivorax*], non-Hodgkin’s lymphoma [cavitary/no species identified], and vaginal verrucous carcinoma [cavitary/ no species identified). Other underlying health conditions included diabetes, alcoholism, malnutrition, elephantiasis, burns, otorrhea and rhinitis, schizophrenia, and senile dementia. Poor personal hygiene and low socioeconomic status was reported in some cases.

### Treatment

Fourteen lesions in thirteen (33%) clinical cases received ivermectin (one topically, two intramuscularly, and the others orally from 200 to 400 μg/kg for 1 to 5 days) often followed by manual extraction. Ivermectin was observed to kill the larvae in all cases. Other treatments included surgical (16 or 41%) and manual extraction (18 or 46%), use of cigarette smoke and mechanical aspiration.

## Discussion

Human myiasis is a recognized but under-reported health problem in Ecuador. Furuncular myiasis is known locally as ‘‘tupe”, “gusano de monte” or “gusano de bijao” while wound and cavity myiasis as “gusanera”. The indigenous Kichwa and Shuar tribes of the Amazon called it “kuruta wachak” and “munai”, respectively. In this review, we have presented data to show that this ectoparasitic infestation is endemic in all four geoclimatic regions of the country including the Galapagos Islands. Although myiasis is generally regarded as a tropical disease, our data show that it is also present in the temperate Andes, primarily among rural populations, although the incidence is greater in tropical regions of the Amazon and Pacific Coast regions. It is likely that incidence is underestimated substantially given that the population at risk has limited access to health facilities, particularly among indigenous groups in the Amazon region, and most patients with furuncular myiasis self-manage the condition using a variety of traditional remedies.

*D*. *hominis* was identified in almost half recorded clinical cases for whom identification was done, and occurred in tropical and subtropical regions of the Coast and Amazon. No cases caused by *D*. *hominis* were reported from the temperate Andes in accordance with the fact that *D*. *hominis* does not survive above an altitude of 1500m [13]. While *C*. *hominivorax* caused myiasis in both tropical and temperate regions, disease caused by this species was primarily identified among indigenous populations living in the Andean region where temperature ranges 8 to 18°C. *C*. *hominivorax* abounds in the foothills of the western Andes up to an altitude of 1,250m where it is the primary cause of bovine and ovine infestations [14], but appears also at higher altitudes during warmer months [13]. Previous studies indicate that *D*. *hominis* and *C*. *hominivorax* are the predominant agents of human myiasis in the New World [15]. Climatic changes due to global warming could modify the distribution of both flies resulting in future infestations in areas that are presently too cold to sustain these fly species.

Here we report for the first time an infestation with the facultative fly, *Lucilia eximia* (Wiedemann, 1819) (Diptera: Calliphoridae), in a prostrate elderly woman with a large necrotic leg wound from the mountainous Andes. The occurrence of *Lucilia* spp infestation has been reported infrequently with most cases from Australia where it is caused by *L*. *cuprina* [13]. Official data from the Ecuadorian MPH did not provide data on causative fly species. Reporting of myiasis within the MPH is not compulsory and the database carries records of diagnoses among outpatients but not of hospitalized inpatients, likely resulting in underreporting. Forty-one different species of dipterous flies have been reported to cause human myiasis worldwide [16], and we believe that it is likely that other species, in addition to the 5 already identified, will be implicated as causes of human myiasis in Ecuador if surveillance of myiasis and capacity for species identification can be improved. Physicians need to work more closely with biologists and entomologists to allow identification of new or invasive species across the diverse geoclimatic regions within the country.

Infestations of wounds and natural cavities with *C*. *hominivorax* resulted in severe disease, often associated with the presence of dozens of maggots in each lesion destroying healthy as well as necrotic tissue and resulting in disfigurement and blindness. Furthermore, *C*. *hominivorax* was implicated in a nosocomial case of myiasis, as reported previously [13], reflecting the capacity of the fly to infest open wounds or cavities in hospitals and nursing homes. Flies of *C*. *hominivorax* were also associated with infestations in the homeless and indigent. Since the fly is able to survive in more temperate regions of the Andes, particularly during warmer months, this puts the capital Quito at an altitude of 2,850 m and surrounding valleys within the area at risk for wound and/or cavitary infestations. *C*. *hominivorax* larvae can invade healthy tissues and burrow into adjacent organs, such as the brain, causing a rapidly fulminating and life-threatening illness [17].

Surgical removal and manual extraction are recommended for furuncular and for wound and some cavitary myiasis, respectively [17]. For wound and cavitary myiasis, tissue debridement and larval removal is often required [12]. In the present review, ivermectin was used in the treatment of fourteen clinical lesions (5 cavitary, 7 wound and 2 furuncular lesions). All individuals treated with ivermectin (200 to 400 μg/kg for one to five days) had their dead larvae (deflated, collapsed and retracted spines) expelled or removed manually within 24 to 48 h. Several studies have shown that ivermectin kills the larvae [17]. Care is required not to damage the larvae on extraction [2] and to ensure all larval debris (after ivermectin treatment) is removed to avoid the risk of secondary infections. Prior to the availability of ivermectin, surgical intervention was the treatment of choice while manual extraction was used for wound and cavitary myiasis. Widely used remedies for furuncular lesions among patients in rural areas are to ‘suffocate’ the larva using cigarette smoke or covering the pore with petroleum jelly, animal fat, nail polish, or topical application of animal vermifuges.

In conclusion, we report a significant annual incidence of myiasis throughout Ecuador, mainly in tropical and subtropical regions of the country but also in more temperate and mountainous regions. Furuncular myiasis caused by *D*. *hominis* was most common, including among travelers, while wound and cavitary myiasis, caused primarily by C. *hominivorax* was observed among the elderly and malnourished. Risk factors included poverty and contact with domestic animals, consistent with studies in neighboring countries [17]. From a travel health perspective, a knowledge of the geographical distribution of myiasis-causing species will improve clinical management. There is a need for greater awareness among and training of health professionals on myiasis, reporting of cases, identification of fly species, and appropriate treatment. Fly larvae found in lesions should be preserved for taxonomic identification.

## Supporting information

S1 Data Myiasis data extracted from Ecuadorian Ministry of Public Health National Automated Registry of Out-Patient Medical Consultations (Registro Diario Automatizado de Consultas y Atenciones Ambulatorias, RDACAA). (XLSX)
